# Supplementary material for: Prop3D: A flexible, Python-based platform for machine learning with protein structural properties and biophysical data
Source: BMC Bioinformatics. 2024 Jan 4;25:11. doi: 10.1186/s12859-023-05586-5 (PMC10768222; doi:10.1186/s12859-023-05586-5)
Supplement: Supplementary file 1 — Additional file 1: Table 1. Sequence-based bioinformatics tools available in Prop3D. Table 2. Structural bioinformatics software suites available in Prop3D. §3. How Prop3D abides by the FAIR guidelines. [file 12859_2023_5586_MOESM1_ESM.pdf]

Supplemental Material for *Prop3D: A Flexible, Python-based Platform for Machine Learning with Protein Structural Properties and Biophysical Data*

Eli J. Draizen[1,2*], John Readey[3], Cameron Mura[1,2*], and Philip E. Bourne[1,2]
[1]Department of Biomedical Engineering,
[2]School of Data Science, University of Virginia, Charlottesville, VA, USA
[3]The HDF Group, Bellevue, WA, USA
[*]Corresponding Authors: E-mail: e.draizen@gmail.com, cmura@virginia.edu

doi:xx.xxxx/xxxx.x.xx.xxxxxxx

September 15, 2023

Contents

1 Sequence-based bioinformatics tools available in Prop3D **2**

2 Structural bioinformatics software suites available in Prop3D **3**

3 How Prop3D abides by the FAIR guidelines **4**

1 Sequence-based bioinformatics tools available in **Prop3D**

Name	Description	Wikidata entry
**BLAST**	Search sequences (or groups of sequences) against the non-redundant (NR) database or a custom database	Q286820
**DeepSequence**	A generative latent variable model for biological sequence families	Q114841036
**ESM**	Pre-trained language models for proteins	Q114841163
**EVcouplings**	Evolutionary couplings from protein and RNA sequence alignments	Q114841016
**HMMER**	Build HMMER models with a group of sequences or at a given level of the CATH hierarchy, search a a group of sequences with a pretrained model, or use jackhmmer starting from a single sequence	Q5631078
**MMSeqs2**	Ultra-fast and sensitive search and clustering suite	Q114840759
**MUSCLE**	MUltiple Sequence Comparison by Log-Expectation (state-of-the-art tool for creating MSAs)	Q6719088
**SeqDesign**	Protein design and variant prediction using autoregressive generative models	Q114841058
**USEARCH**	High-throughput sequence search and clustering analysis tool	Q114841186

Table 1: **Sequence-based bioinformatics tools available in Prop3D**. Most of these tools have been dockerized, and are available at our Docker Hub (https://hub.docker.com/u/edraizen).

2 Structural bioinformatics software suites available in Prop3D

Name	Description/purpose (in this context)	Wikidata entry
**APBS**	Adaptive Poisson-Boltzmann Solver, used here to calculate the electrostatic potential for each atom in a given protein	Q65072984
**Consurf**	Get pre-calculated conservation scores	Q112888886
**CNS**	Energy minimize a given structure	Q5191443
**CX**	Get curvature for each atom in a given protein	Q114841750
**DSSP**	Calculate secondary structure and accessibility for each residue in a given structure	Q5206192
**EPPIC**	Calculate sequence conservation scores for a given protein and obtain biologically relevant protein interactions (i.e., not resulting from crystal packing)	Q114841783
**foldseek**	Fast searching and clustering of protein structure databases	Q114840749
**FreeSASA**	Get solvent accessibility of each atom in a given protein	Q114841793
**Geometricus**	A structure-based, alignment-free embedding approach for proteins, utilizing moment invariants	Q114840743
**HADDOCK**	Dock two proteins or refine the conformation of two docked proteins	Q114841798
**MaxCluster**	Cluster very similar structures	Q114840623
**MGLTools**	Convert atom names to AutoDock names and PDBQT	Q114840701
**MM-Align**	Align two protein complexes	Q114841843
**mTM-Align**	Multiple structure alignment	Q114841813
**MODELLER**	Create full atom structures from C_α only models, mutate structures with different amino acids, 'remodel structure' to energy minimize, and model loops	Q3859815
**MSMS**	Calculate molecular surfaces and create meshes	Q114841806
**Multivalue**	Merge electrostatic values from multiple atoms, e.g. on a protein surface	Q114840933
**OpenBabel**	Convert to PDBQT format for AutoDock atom naming	Q612752
**PDB2PQR**	Protonate a protein structure, debump hydrogens, energy-minimize, and standardise naming (atomic nomenclature)	Q62856803
**pdb-tools**	A "Swiss army knife of tools" to manipulate PDB files	Q114840802
**PRODIGY**	Predict binding affinties (K_D) and "fraction of common contacts" in complexes	Q114840854
**REDUCE**	Protonate and de-protonate structures	Q114840896
**SCWRL4**	Correct side-chains using the Dunbrack rotamer library	Q114840881
**TM-Align**	Align two or more protein 3D structures	Q114840775

Table 2: **Structural bioinformatics software suites available in Prop3D**. Most of these tools have been dockerized, and are available at our Docker Hub (https://hub.docker.com/u/edraizen).

3 How Prop3D abides by the FAIR guidelines

In general, the creation of scalable, reproducible scientific workflows faces challenges that stem from the sheer volume and heterogeneity of available data-sources and data-types, in addition to potential other factors such as the variable range of computing platforms, architectures and capabilities that one may seek to deploy a workflow across (e.g., multi-core processing on a local workstation, versus a Linux HPC cluster, versus an eScience grid or other highly distributed network environment in the cloud). The 'FAIR' principles for scientific data provide a set of best-practices that contribute to the research enterprise by striving to make datasets *Findable*, *Accessible*, *Interoperable*, and *Reproducible*. In other words, FAIR datasets should be (i) easy to *find*, with appropriate metadata to facilitate searching by others; (ii) one should be able to *access* all of the data easily, without undue effort; (iii) one should be able to integrate and otherwise *interoperate* the data with other data-sources and software frameworks; and (iv) the data should be (re)usable and *replicable* by others (a bedrock of the scientific method). When possible, these guidelines (`https://www.go-fair.org/fair-principles`) would apply equally well to both the datasets themselves as well as to the code that underlies the data-generating and data-processing/analysis/reduction pipelines—i.e., the software framework would be FAIR-compliant, insofar as its resultant data are FAIR. The following enumerates how Prop3D complies with these guidelines:

1. **Findable**

   The first step in (re)using data is to be able to find it. Metadata and data should be easy to find for both humans and computers. Machine-readable metadata are essential for automated discovery of datasets and services, so this is an essential component of the FAIRification process. In Prop3D, the following hold:

   F1. (Meta)data are assigned a globally unique and persistent identifier

   F2. Data are described with rich metadata (defined by R1 below)

   F3. Metadata clearly and explicitly include the identifier of the data they describe

   F4. (Meta)data are registered or indexed in a searchable resource

2. **Accessible**

   Once a user finds the required data, she/he/they need to know how such data can be accessed, possibly including issues of authentication and authorisation. In Prop3D,

   A1. (Meta)data are retrievable by their identifier using a standardised communications protocol

   A1.1 The protocol is open, free, and universally implementable

   A1.2 The protocol allows for an authentication and authorisation procedure, where necessary

   A2. Metadata are accessible, even when the underlying data are no longer available

3. **Interoperable**

   Datasets generally are not used in a vacuum, and at some point or another will need to be integrated with other types and sources of data. In addition, the data need to interoperate with available applications or workflows for analysis, storage, and processing. In Prop3D,

   I1. (Meta)data use a formal, accessible, shared, and broadly applicable language for knowledge representation.

   I2. (Meta)data use vocabularies that follow FAIR principles

   I3. (Meta)data include qualified references to other (meta)data

4. **Reusable**

   The ultimate goal of FAIR is to optimise the reuse of data. To achieve this, metadata and data should be well-described so that they can be replicated and/or combined in different settings. In Prop3D,

   R1. (Meta)data are richly described with a plurality of accurate and relevant attributes

   R1.1. (Meta)data are released with a clear and accessible data usage license

   R1.2. (Meta)data are associated with detailed provenance

   R1.3. (Meta)data meet domain-relevant community standards
